# The study of sirtuins in breast cancer patients before and after radiotherapy

**DOI:** 10.3906/sag-2012-195

**Published:** 2021-06-28

**Authors:** Nazlı HELVACI, Hatice SARAÇOĞLU, Oğuz Galip YILDIZ, Eser KILIÇ

**Affiliations:** 1 Health Sciences Institute, Erciyes University, Kayseri Turkey; 2 Department of Medical Biochemistry, Faculty of Medicine, Erciyes University, Kayseri Turkey; 3 Department of Radiation Oncology, Faculty of Medicine, Erciyes University, Kayseri Turkey

**Keywords:** Sirtuin, breast cancer, radiotherapy

## Abstract

**Background/aim:**

Targeting the new and unique proteins is an important medical strategy for treating breast cancer. It is quite important to find out proteins that have a role in the development of cancer. Sirtuins (SIRT) are well related in different physiological activities and connected with cancer. We aimed to determine the effect of radiotherapy on SIRT1 and SIRT2, which have not been yet been clarified as a tumor suppressor or promoter.

**Materials and methods:**

Twenty-two women with nonmetastatic breast cancer enrolled in the study. Blood samples were taken before and after radiotherapy, soluble SIRT1 and SIRT2 levels were determined with ELISA kits.

**Results:**

There was no difference in SIRT1 levels before and after radiotherapy (p = 0.548). SIRT2 levels were significantly found to be decreased after radiotherapy (p = 0.042). There was a strong and positive correlation before radiotherapy (p < 0.001), and a moderate and positive correlation after radiotherapy (p = 0.007) between SIRT1 and SIRT2.

**Conclusion:**

These results suggest that SIRT2 may provide a new strategy for follow-up of breast cancer treatment. Additionally, by emphasizing the importance of SIRT2 in breast cancer, it opens ways to provide grounds for the development of the next generation of SIRT2-specific radiotracers. Finally, the most important thing, in fact, the positive correlation between SIRT1 and SIRT2 both before and after radiotherapy, appears to be clear evidence suggesting more oncogenic roles of sirtuins.

## 1. Introduction

Breast cancer is one of the most important cancer types in the world. It is a complex, multifactorial disease with genetic and environmental factors. It is well known that one of the main risk factors for breast cancer is a family history. At the same time, some other nongenetic risk factors affect disease etiology [1].

Radiotherapy plays a major role in the treatment of nonmetastatic breast cancer. Approaches in medical oncology have resulted in radiotherapy as a potent treatment in the routine administration of most stages of breast cancer [2,3].

Targeting new and unique proteins is an important medical strategy for treating breast cancer. It is quite important to find out proteins that have the role as a promoter or suppressor in the development of cancer. 

Sirtuins (SIRT) are well related in different physiological activities and therefore connected with cancer. SIRTs are known as coenzyme NAD+-dependent histone deacetylases for the movement of altered acetyl groups. The SIRT family has emerged as important regulators of various biochemical events. Novel works exhibit that fixed on shutting up of SIRT1 appearance or action by the removed in breast cancer 1 (DBC1) may be valuable by encouraging p53-induced apoptosis in cancer and by sensitizing cancerous cells to radiation treatment [4,5]. 

In human cancers, it is presently unclear whether increased SIRT1 or SIRT2 expression irritates or obstructs the forming and/or preservation of malignancy. Studies showed that SIRT1 and SIRT2 might be anticipated to assist tumor triggering, growth, and drug resistance. Conversely, some studies propose that SIRT1 and SIRT2 themselves can attend as a tumor suppressor, at least in particular circumstances. Among its activities, for example, SIRT1 and SIRT2 have a crucial role to promote DNA damage restoration action [6–9].

Therefore, the dual role of (promotor or suppressor) SIRT1 and also SIRT2 in modulating the tumor, makes them very attractive. 

The target of this study was to find the consequence of radiotherapy on soluble SIRT1 and SIRT2 proteins, which have not been yet clarified as a tumor suppressor or promoter molecule. 

## 2. Materials and methods

From January 2019 till June 2019, patients admitted to the Radiation Oncology Department in Erciyes University Medical Faculty for the reason of treatment were requested to consider joining the clinical research.

Twenty-two women with nonmetastatic breast cancer **(** stages 1 or 2 **)** enrolled in the study. All patients, after 2 weeks of hormone therapy with no other chemotherapy (trastuzumab), treated with radiotherapy. 8 (%36) of the patients took irradiation of the breast, 7 (%32) of the chest wall, 4 of the breast and axilla (%18), 3 (%14) of the chest wall and axilla** [conventional dose, 50 Gy + 10 Gy (boost) at 2 Gy/day on the breast and 50 Gy at 2 Gy/day on the regional lymph nodes or chest wall) for six weeks after the adjuvant hormone treatment]. Intensity-modulated radiation therapy (IMRT) plan was used.

Blood samples were collected prospectively from patients just in advance on the day of starting the radiation therapy (after 2 weeks of finishing hormone therapy) and immediately after the last dose of radiation therapy. The timing of the blood withdrawals was designed to coincide with scheduled vein punctures and clinic appointments to prevent patients from having additional clinic visits or blood sample withdrawals merely for research aims. 

Inclusion criteria were no history of serious illness, have no chronic disease such as; cardiovascular or pulmonary disease or diabetes mellitus, have no cancer diagnosis, >18 years old, and nonsmokers. 

All blood samples (˜3 mL) were collected in tubes and the samples were centrifuged for serum removal. Blood samples were centrifuged at 2000 x g for 10 min, and only the top 1 mL of serum supernatant was removed to prevent contamination. The serum samples were kept frozen at –80°C until assayed for soluble SIRT1 and SIRT2 protein levels.

Serum concentrations of soluble SIRT1 and SIRT2 were determined with commercially present enzyme-linked immunosorbent assay (ELISA) kits, using a sandwich ELISA technique (SunRed bio, Shanghai, PR China, Catalogue numbers: 201-12-2558 for SIRT1 and 201-12-2559 for SIRT2). Assays were carried out according to the manufacturer’s instructions. 

The sensitivities of each assay were 0.306 ng/mL for SIRT1 and 0.147 ng/mL for SIRT2. The inter-and intra-assay coefficients of variation were CV < 12% and CV < 10%, for SIRT2, and also the assay range for SIRT1 and SIRT2 were 0.5 **–** 40 ng/mL and 0.2 **–** 60 ng/mL respectively.

The ethical and methodological aspects of this investigation were approved by the Institutional Review Board of Erciyes University Medical Faculty. Written informed agreements were supplied by the subscribers to enter in this study. We confirm that all methods were performed by the proper instructions and arrangements.

All data were entered into a database. Statistical analysis was performed using the TURCOSA software Turcosa Analitik Ltd Co, Türkiye . Online www.turcosa.com.tr [accessed on 21.06.2019]. 

The comparison between pre-and post-radiotherapy groups in serum soluble SIRT1 and SIRT2 was carried out using the Wilcoxon test and the correlation analysis was done using the Spearman test. A p < 0.05 was assessed statistically significant.

## 3. Results

**Figure 1:**
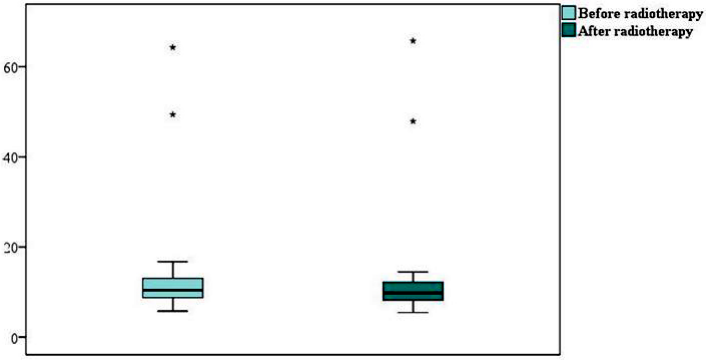
A graph showing serum levels of soluble SIRT1 levels before and after radiotherapy. There was no statistically significant difference in soluble SIRT1 protein levels before and after radiotherapy (p = 0.548).

**Figure 2:**
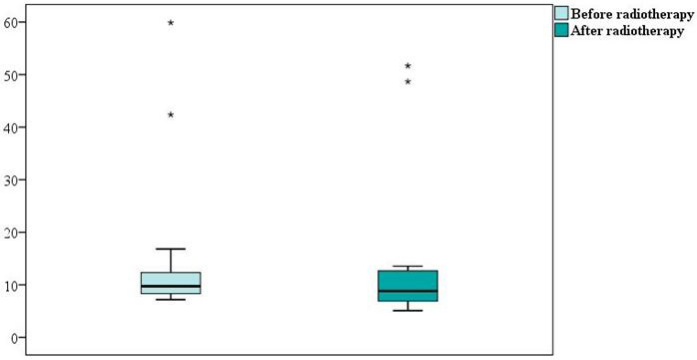
A graph showing serum levels of soluble SIRT2 levels before and after radiotherapy. There was statistically clear significant difference in soluble SIRT2 protein levels before and after radiotherapy (p = 0.042).

**Figure 3:**
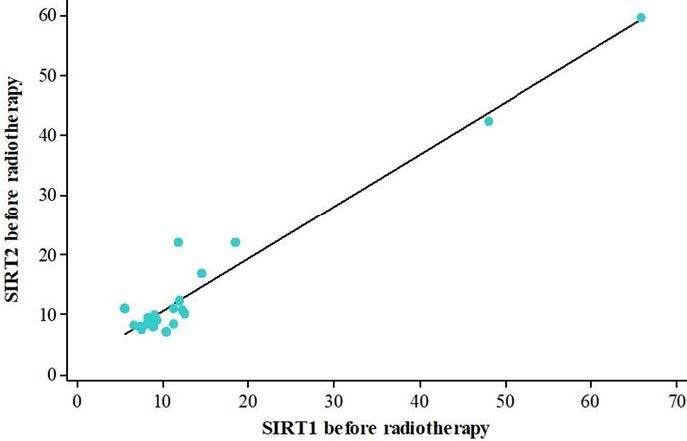
A strong, positive, and statistically significant correlation between soluble SIRT1 and SIRT2 proteins before radiotherapy (p < 0.001).

**Figure 4:**
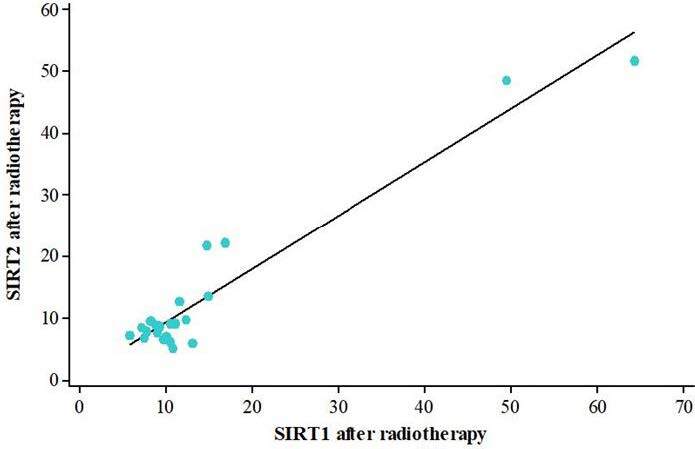
A moderate, positive, and statistically significant correlation was found between soluble SIRT1 and SIRT2 proteins after radiotherapy terminated (p = 0.007).

There was no statistically significant difference in soluble SIRT1 protein levels before and after radiotherapy (p = 0.548) (Figure 1). Soluble SIRT2 protein levels were significantly found to be decreased after radiotherapy treatment (p = 0.042) (Figure 2). There was a strong, positive, and statistically significant correlation between soluble SIRT1 and SIRT2 proteins before radiotherapy (p < 0.001) (Figure3), and a moderate, positive, and statistically significant correlation was found between soluble SIRT1 and SIRT2 proteins after radiotherapy terminated (p = 0.007) (Figure 4).

## 4. Discussion

The researches on the expression of SIRTs suggests that regulation of their activity could have positive results in supporting the treatment of certain metabolic or cancer diseases. SIRTs can also be prognostic markers of some pathological conditions. SIRTs are found in all living organisms and regulating histone acetylation. They are taken roles in many other processes, including involvement in the regulation of the cell cycle and also in the process of cell differentiation and apoptosis [10]. 

In mammals, seven SIRT homologs (SIRT1-7) are present. SIRT1 and SIRT2 are extensively spread in humans and controls biological functions. Additionally, they may have a very important task in cancer [11–13]. Earlier works have found that the unusual quantity of SIRT1 and SIRT2 were related to a wide range of malignant tumors. They have diverse roles in varied cells and possess a significant role in tumorigenesis (both tumor-promoting and tumor-suppressing function). However, the mechanisms in which they have the roles in cancer are still controversial [14]. 

Based upon the monitoring that SIRT1 is regulated and changed in malignant growth of the cell, the assumption is that SIRT1 may encourage tumorigenesis by modifying cellular signaling. SIRT1 was assumed to be a likely tumor promoter since it is negatively arranged mainly p53 and other tumor suppressors. SIRT1 also behaves as a tumor suppressor based upon its character in down-regulating survivin and β-catenin. The several functional characters of SIRT1, particularly the argument for both tumor promoter and tumor suppressor roles, have given glamor attention in SIRT1 for cancer treatment [15–17]. 

On the other hand, although it has been known that SIRT2 acts as a major character in carcinogenesis, whether it has been attributed to both tumor-promoting and tumor-suppressing activities is unclear. As a result, while the functions of SIRT2 in tumorigenesis have been reported and reviewed in many studies, the connection and mechanism between SIRT2 and the cancer are less established [18,19].

As of now, it would be good to report tumor and treatment features that may affect some SIRT protein expressions, especially relating to radioactivity. For example; it is known that SIRT 6 can rise the radiosensitivity of nonsmall cell lung cancer and have preventive roles on radiation-induced lung injury [20]. 

In addition, miR 125a-5p could increase SIRT7 and also raised apoptosis in lung cancer cells to rise their radiosensitivity and miR-22 suppresses cancerogenesis and enhances radiosensitivity of breast cancer cells by selecting SIRT1, supplying a hopeful therapeutic objective for breast cancer [21,22].

It has been also shown that 5-fluorouracil stimulates radiosensitivity via SIRT7 breakdown to beneficence the cell death pathway in cancer cells. Thus, the reduction of SIRT7 might be an important action to raise the influence of chemoradiation therapy in cancer patients [23].

One of the latest research also showed a new duty for SIRT3 in oral carcinogenesis as a promoter of cell proliferation and survival, therefore suggesting SIRT3 as a novel possible therapeutic objective to treat oral cancer [24].

Studies have demonstrated that SIRT1 and SIRT2 can raise the capacity of tumor cells to break through the membrane, letting tumor cells get extremely transformed and so much invasive capacity. In breast cancer cells, SIRT2 could stop the peroxiredoxin-1 activity, increase the responsiveness of breast cancer cells to reactive oxygen species stimulated DNA damage and so encourage the apoptosis of cancer cells [25].

As we have mentioned earlier in the manuscript and is known that there is emerging evidence of a role for several sirtuins in carcinogenesis. However, to our knowledge, their role in breast cancer has not been investigated previously. It is also known that SIRT1 and SIRT2 proteins play an important role in the tumor cells, especially during chemotherapy. However, there is no clear evidence of radiotherapy’s effect on these protein levels in cancer [26,27]. 

In the present study, we tried to explore the potential role of radioactivity by evaluating expression protein levels of SIRT1 and 2 in breast cancer patients. In other words, this present article discusses whether radioactivity has an activating or inhibiting effect on individual SIRT1 and SIRT2 proteins in breast cancer.

It is known that SIRT1 could operate stress and DNA maintenance procedures, thus letting the conservation of the genomic unity. Contrarily, it has also been shown that SIRT1 overexpression can magnify tumor enlargement and support cell endurance in response to drug resistance and stress. Even so, SIRT1 is regulated differently in the scope of tumors involving breast carcinoma [26–28].

Our data suggest that radiotherapy reduced SIRT2 level and this reduced level in soluble proteins may be useful in cancer therapy. In other words, it is possible that especially SIRT2 or may be SIRT1 suppression could be influential in treating particular available or preexisting cancers, classically and probably via up-organizing p53-mediated apoptosis. 

Over observation of SIRT2 could remarkably extend the mitotic (M) phase and retardate mitotic exit. As a result, it has been nominated that SIRT2 may responsible as a mitotic checkpoint protein in G2-M to stop the initiation of chromosomal imbalance, especially in reaction to microtubule inhibitor-mediated mitotic stress. Therefore, tumors with earlier stages could be sensitive to radiotherapy or chemotherapy via SIRT2 proteins.

With our present results, we could clearly say that down-regulation of soluble SIRT2 level in human breast cancer with radiotherapy may be affected to stop or at least slowdown tumorigenesis.

As it is seen in the present study of the influence of radiotherapy which adjusts soluble SIRT1 and SIRT2 levels (positively or negatively) seems to be important to develop novel effective drugs against tumors. As both SIRT1 and SIRT2 are considerable organizers of cellular old aging, upwards investigation on SIRT1-SIRT2 signaling will presumably supply particular indications for the appreciation of the more difficult connections among cellular old aging and tumorigenesis.

This work gives new information on the expression of SIRT1 and SIRT2 levels in individuals in the case of radiotherapy in breast cancer patients. Therefore, it shows biological roles that SIRT1 and SIRT2 play in the human organism in connection with radioactivity therapy during breast cancer treatment. Our findings of SIRT2 expression due to radioactivity in breast cancer was compared with SIRT1 expression showed that SIRT2 may clearly play an important role in breast cancer.

It is important to not forget that these and other previous findings by others are all contradictory due to SIRT proteins specialty; however, it must be also noted that these studies were limited because they are only either related to gene array or protein expression and did not include the kinds of full functional assessment of SIRT’s in vitro or in vivo.

However, we could conclude from this study that the down-regulation of SIRT2 after radiotherapy could probably induce apoptosis in breast cancer. This may implicate SIRT2 as a new potential therapeutic target for treating breast cancer. Additionally, these results suggest that SIRT2 may provide a new strategy for follow-up of breast cancer treatment.

Finally, it should not be forgotten that SIRT2 has been identified as having both cancer-promoting functions (i.e., through stabilization of Myc oncoproteins in breast cancer, as well as cancer-suppressing functions (i.e., through tubulin regulation)_[29]. Therefore, the development of invasive or if possible noninvasive molecular imaging approaches for monitoring SIRT2 could be a very useful tool for breast cancer.

Additionally, by emphasizing the importance of SIRT2 in breast cancer, we could open ways to provide grounds for the development of the next generation of SIRT2-specific radiotracers.

As it was also observed with the present study and the debatable duties of SIRT1 and SIRT2 in cancer; it could be thought that more examination on the appearance proportion of soluble SIRT1 and SIRT2 proteins in human (breast) cancer needs to be done. Additionally, the works of some molecules will also assistance to figure out the mechanism underlying the fundamental biological role of SIRT1 and SIRT2 proteins. Forming of transgenic animals with overexpression or down-regulation of SIRT1 and SIRT2 proteins for tumorigenesis evaluation, research of the influence of other targets of SIRT1 and SIRT2 such as different doses or durations of radiations on interactions among SIRT1/SIRT2 and p53 in human cancers could be other targets.

Cancer continues to prove complex and multifaceted. As our understanding and knowledge of the initiation, progression, and therapy of malignant tumorigenesis progress, it seems that it is likely to deepen in cancer with new targets.

## Informed Consent

The ethical and methodological aspects of this investigation were approved by the Institutional Review Board of Erciyes University Faculty of Medicine. Written informed agreements were supplied by the subscribers to enter in this study.
